# Long term outcomes of the Humeral Intracondylar Repair System for management of canine humeral intracondylar fissures and humeral condylar fractures

**DOI:** 10.3389/fvets.2023.1296940

**Published:** 2024-01-03

**Authors:** Rebecca S. Hood, Myles Ben Walton, John F. Innes

**Affiliations:** ^1^ChesterGates Veterinary Specialists CVS Group plc, Chester, United Kingdom; ^2^Small Animal Teaching Hospital, University of Liverpool Leahurst Campus, Neston, United Kingdom; ^3^Movement Referrals: Independent Veterinary Specialists, Runcorn, United Kingdom

**Keywords:** humeral intracondylar fissure, IOHC, incomplete ossification of the humeral condyle, fracture, surgery, elbow, dog

## Abstract

**Objective:**

To document long-term client-reported clinical outcomes and complications for the Humeral Intracondylar Repair System (HIRS) for treatment of humeral intracondylar fissures (HIF) and humeral condylar fractures (HCF) in dogs.

**Method:**

Data collection involved the review of clinical records and analysis of an owner questionnaire regarding complication occurrence and client-reported outcome. The “Liverpool Osteoarthritis in Dogs” (LOAD) instrument was incorporated into the questionnaire.

**Results:**

Twenty-six cases of HIF and 14 cases of HCF were included in the study, with follow-up times of over 12 months (range 13–97 months). Thirty-seven out of 40 cases reached long-term follow up: 25 out of 26 HIF cases, 11 out of 11 lateral condylar fracture cases and one out of three dicondylar fracture cases. Two cases of HIF suffered a gradual return of lameness in the long term; both dogs had concomitant medial coronoid disease. No other complications were reported in the long term. Excluding cases with concurrent issues affecting exercise, the median LOAD score at follow-up was 4 and 5 (out of 52) for HIF and HCF cases, respectively. At long-term follow-up, 36 out of 37 cases were reported to have regained “full function of the limb.”

**Clinical significance:**

The results of this study, together with previously reported short and medium-term outcomes, support the use of HIRS for management of humeral intracondylar fissures and humeral condylar fractures.

## Introduction

Humeral intracondylar fissure (HIF), previously termed incomplete ossification of the humeral condyle (IOHC), is a sagittal fissure of the humeral condyle, which may be complete or partial (1). HIF weakens the condyle and predisposes to humeral condylar fractures (HCF). HIF can also cause lameness and elbow pain (2). Previously, all HIFs were thought to be a manifestation of incomplete ossification of the humeral condyle but reports of *de novo* HIF formation and propagation of partial HIF to complete HIF, along with the sparse reports of histologic analysis have supported the hypothesis that many HIFs are a stress fracture (1–8). However, our understanding of the full etiopathogenesis of HIF remains incomplete (9).

A proportion of HIFs propagate to HCF, with one study reporting that 18% of clinically silent, conservatively managed HIF cases progressed to HCF at a mean of 14 months after diagnosis, and overall, 24% of cases required surgery after the initial diagnosis, either due to fracture or progressive lameness (10). Another study reported that 43% of dogs presenting with a unilateral condylar fracture had computed tomography (CT) confirmation of HIF in the contralateral humerus (11).

Surgical management of HIF aims to ameliorate pain, lameness, and to protect against HCF. Most commonly a transcondylar implant is placed and this has occasionally been combined with efforts to improve the chances of healing either through forage or combination with bone autograft (2, 7, 12). Surgical management has been associated with unacceptably high complication rates, reported up to 60% in some studies, with seroma formation and surgical site infection (SSI) being the most common (13–16). These reports indicate that there is a need for a method to treat clinically evident HIF, and act as a prophylactic measure against HCF, which does not involve such a high risk of complications. The Humeral Intracondylar Repair System (HIRS^TM^)^I^ is an implant and associated instrumentation that was specifically designed for treatment of HIF. The design and surgical technique for HIRS placement, and short to medium-term outcomes have been reported previously, with a major complication rate of 6% for HIF treatment and 21% for HCF repair (17). Here, we present the long-term clinical outcomes and complication rates.

## Materials and methods

### Study design

Ethical approval was granted by the CVS Group plc Ethics Review Board. Long-term follow-up was defined as “over 12 months post-operatively,” as advised by Cook et al. (20). “Clinically evident HIF” was defined as HIF that was associated with lameness and “clinically silent HIF” was defined as “HIF identified by diagnostic imaging on contralateral elbows without any evidence of elbow pain.” The database at a single referral center was searched for cases of HIF or HCF treated with HIRS over 12 months before the study start date. Owners were contacted via email and post to obtain consent for their animal's inclusion in the study and to complete a questionnaire, which was followed up with a telephone call if no response had been received within 3 weeks. Clinical records were reviewed for data regarding the surgical procedure, prophylactic antibiosis and the incidence of short-term complications. This data was utilized to identify cases for which the outcome had been unsuccessful, i.e., the HIRS was no longer in place, either due to removal, amputation, or euthanasia. Owner consent was obtained for the inclusion of their animal's clinical data in the study; no questionnaire was issued as long-term assessment of the HIRS implant could not be performed.

All other cases were contacted to gain consent for clinical data use and to complete a questionnaire. The questionnaire was designed to answer specific questions regarding the long-term outcome of surgery and included sections on incidence of complications in both the short and long term, as reported by the owners. A nested “Liverpool Osteoarthritis in Dogs” (LOAD) questionnaire was also incorporated to provide a validated client-reported outcome measure. A higher LOAD score indicates a greater level of owner-perceived disability, with a range of 0–52 possible (18). Details of the questionnaire, excluding the copyrighted LOAD questions, are provided in the Supplementary material.

### Surgery

All cases were operated at the same center. In all HIF cases, a medial approach to the humeral condyle was performed and the HIRS was placed in a medial-to-lateral direction as previously described (17). In order to position and direct the pilot hole for HIRS placement, surgeons used a variety of methods including the use of custom 3D-printed aiming devices, fluoroscopic guidance, CT planning and proprietary aiming devices, and free-hand aiming. Implant positioning was assessed by intra-operative fluoroscopy and/or post-operative radiographs. The void around the central threadless portion of the HIRS screw was packed with demineralized bone matrix putty ^II^ before the HIRS was fully advanced.

Lateral condylar fractures (LCF) were approached via a lateral approach and dicondylar fractures (DCF) were approached via medial and lateral approaches. For HCF cases, normograde and retrograde (inside-out and outside-in methods) were used to create the pilot hole for the HIRS drill bit. The fracture was reduced and the HIRS placed across the intracondylar fracture (19). In LCF cases, the HIRS implant was placed in a lateral-to-medial direction. In DCF cases, the HIRS was placed in either a medial-to-lateral or lateral-to-medial fashion. LCF cases were stabilized with the HIRS and either an epicondylar locking compression plate (7/11), a Lateral Epicondylar Anatomical Plate (LEAP^TM^)^III^ (3/11) or lag screw (1/11). All DCFs were stabilized with the HIRS and bilateral epicondylar plates. The LEAP^TM^ was used on the lateral aspect in two DCF cases.

Peri-operative antibiotics were administered in all cases, with the decision to use post-operative antibiotics determined by surgeon preference. 38/40 cases (95%) received post-operative antibiotics (cephalexin)^IV^ for varying durations, up to a maximum of 14 days.

## Results

### Cases

#### HIF cases

A total of 26 HIF cases were included in the study, 10 of which were included in study on short-term outcomes of the HIRS implant by Walton and others (17). For 25 out of 26 cases (96%) the HIRS implant was *in situ* at follow up and their long-term outcomes were assessed. Twenty-one out of 25 cases were clinically evident HIF and four cases were clinically silent. The mean and median follow-up times were 44 and 36 months, respectively (range 13–97 months). Seventeen out of 25 cases were Springer Spaniels, with other Spaniel breeds accounting for a further seven cases. The remaining case was a cross breed. Two cases were female (one neutered, one entire) and 23 cases were male (three neutered, 20 entire).

One case of HIF did not reach long-term follow-up due to implant loosening occurring at 2 months post-operatively. The HIRS was replaced with a 3.5 mm transcondylar screw that was placed in lag fashion in a lateral-to-medial direction proximal to the previous drill hole. The HIRS void was filled with demineralized bone matrix, and a lateral plate was also placed for additional support because the contralateral limb had fractured previously; this case and complication was included in Walton and others (17). This was a case of clinically evident HIF in a male cross breed dog.

#### HCF cases

A total of 14 HCF cases were included in the study, consisting of 11 LCF and three DCF. Six of these cases, all LCF, were included in Walton and others (17). All LCF cases had the HIRS implant *in situ* at follow up and one out of three DCF reached long-term follow-up. The mean follow-up time was 54 months and median 56 months (range 16–91 months). Similar breeds were represented as the HIF cases, with Springer Spaniels accounting for the majority (nine out of 12), and other Spaniel breeds the next most frequent (two out of 12). The remaining case was a Border Collie. Nine cases were male entire; two cases were female neutered, and one case was female entire.

Two out of three DCF cases were excluded from long term outcome evaluation due to catastrophic complications as defined by Cook et al. (20). Both were male springer spaniel dogs. One DCF case suffered from a persistent SSI, which ultimately resulted in amputation because revision surgery was considered to carry a guarded prognosis. The other case suffered from loosening of plate screws and revision surgery was performed 1 month post-operatively. Radiography performed 5 months later, due to a sudden deterioration, revealed loose screws in the lateral plate, a broken screw in the medial plate and evidence of osteolysis in the condyle and epicondyle. Euthanasia was performed 6 months after the initial surgery.

### Clinical outcome

#### HIF cases

According to owners, 24 out of 25 cases reaching long-term follow-up returned to “full function of the limb.” Six cases returned to full function “within 1 month of surgery,” 15 “between 1 and 3 months,” two “between 3 and 6 months', and one case “over 6 months” after surgery.

Of 25 cases, owners of 19 reported that their dog never required any analgesics in the long term. Three out of 25 required analgesia less than once monthly, and one case required analgesia multiple times a month, but not every week. Two cases required analgesia every day.

#### HCF cases

Twelve out of 12 HCF cases included in long-term follow-up regained “full function of the limb” according to the owner. One case returned to full function “within 1 month of surgery,” five cases “between 1 and 3 months,” three cases “between 3 and 6 months,” and three cases “over 6 months” after surgery.

Eleven out of 12 cases never required any analgesics in the long term. One case required analgesia multiple times a month but not every week. No owners reported that any cases required analgesia at a more frequent rate.

### LOAD score at follow-up

#### HIF cases

**Figure 1:**
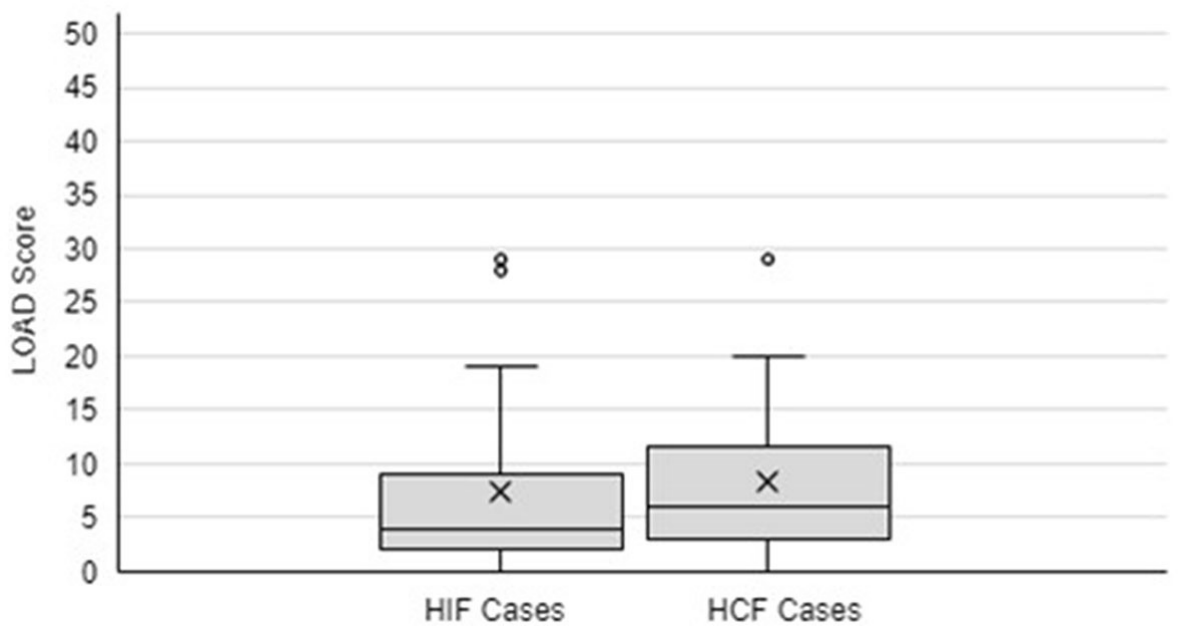
Box and Whisker diagram to demonstrate LOAD Score at follow-up for HIF and HCF cases. X symbolizes the mean. The box symbolizes the interquartile range of the data, with the middle line indicating the median. Outliers are identified by separate circles outside the range of data.

Follow-up LOAD scores were calculated for the 25 HIF cases with questionnaire answers. The mean and median LOAD scores at follow-up were “7” and “4”, respectively (range 0–29), which are within the mild category (18). As shown in Figure 1, outliers skewed the mean in both HIF and HCF populations. Six HIF cases were reported to have concurrent issues that could impact their exercise tolerance and reported mobility, with reports of “elbow osteoarthritis” (1), “hindlimb osteoarthritis” (1), “bilateral cruciate disease and previous Tibial Plateau Leveling Osteotomy performed” (2), “contralateral DCF” (1), and “amputation of the contralateral forelimb” (1). When excluding these cases with concurrent issues, the mean and median LOAD scores at follow-up were “6” and “4”, respectively.

With a cut off LOAD score of “10”, 20 out of 25 cases (80%) were classified as having very mild effect on mobility long term. Excluding those with concurrent conditions that affect their mobility, 17 out of 19 cases (90%) had a LOAD score <10.

#### HCF cases

Follow-up LOAD scores were calculated for the 12 HCF cases with questionnaire responses, which consisted of 11 LCF and 1 DCF. The mean and median LOAD score for dogs at follow-up after surgery were “8” and “6”, respectively (range 0–29). Two cases were reported to have concurrent issues that could impact their perceived mobility, with reports of “elbow osteoarthritis” (1) and “mitral valve disease” (1). Excluding these cases, the mean and median LOAD scores at follow-up were “6” and “5”, respectively, which is within the mild category (18). With a cut off LOAD score of 10, nine out of 12 cases (75%) were classified as a mild effect on mobility. Excluding cases with concurrent conditions that affect their mobility, eight out of 10 cases (80%) had a LOAD score <10.

There was not a significant difference in the likelihood of HCF cases to return to a LOAD score below 10 compared to HIF cases (Fisher's exact test, *p* = 1.0). A Pearson Correlation Coefficient calculation revealed no correlation between age at follow-up and LOAD score for HIF and HCF cases, giving an *r*-value of −0.097, which was not significant at a 95% significance level (*p* = 0.57). Using a two tailed *t*-test with a 95% confidence interval demonstrated no difference in LOAD scores between those cases which suffered from complications in the short term and those that did not (0.955 <2.03).

### Long term complications

Two HIF cases reported a gradual return of lameness in the ipsilateral limb “over 12 months” post-operatively. Both were originally cases of clinically evident HIF with concurrent medial coronoid process disease (MCD) in the same elbow. One case had suffered from a DCF in the contralateral elbow, which had been operated on, and then was treated with the HIRS for HIF. The owner reported this case never regained full function of the limb and required analgesia every day. This case had been euthanized for reasons unrelated to the elbow 3 years post-operatively. The other case suffered from an SSI in the short-term post-operatively, which resolved with antibiotic treatment. The owner reported that this case did return to full function of the limb but required analgesia less than once monthly. They returned for a consultation to investigate this return of lameness. This case had suffered from a non-displaced fragmented medial coronoid process in the same limb 3 years prior to the HIF diagnosis, which was confirmed with CT and managed conservatively. It was concluded that the most likely cause of the lameness was osteoarthritis of the elbow, given the history of fragmented medial coronoid process and the clinical pattern of worsening after rest, and not exacerbated by jumping. However, CT would be required to be certain of implant integrity and this investigation was declined by the owner. All other cases in both HIF and HCF populations reported that no complications occurred at any point over 1 year post-operatively.

## Discussion

The population of cases included in our study is typical of the main breeds affected by HIF and HCF in the UK. It was reported that HIF was identified in 14% of non-lame, non-painful, English Springer Spaniels when they were examined by CT for other clinical conditions (21). The cohort of dogs in the study reported herein is typical of the population that the HIRS will be used in, as most cases in the study were HIF occurring in Spaniel breeds. The HIRS implant was designed based on biometric data from the humeral condyles of HIF-affected English Springer Spaniels. Initially, it was available at a length of 32 mm, and is now available in three lengths (32, 34, and 36 mm). Therefore, HIRS is not suitable for use in small dogs and some larger breeds.

Three cases did not reach long-term follow-up due to major complications occurring in the short term. In one case the HIRS loosened and was replaced with a transcondylar screw. The other two cases were DCF, which are known to be associated with a higher risk of complications. Major surgical complications occurring in DCF repair have been reported as 22–35% (22–25). Due to the extremely small sample population of DCF cases in our study it is not possible to draw meaningful conclusions. As the HIRS becomes more widely used and there is a larger sample population, a better estimate of complications in DCFs repaired using the HIRS will be forthcoming.

Owners reported excellent long-term outcomes, with 36 out of the 37 cases that reached long-term assessment “regaining full function of the limb” after surgery, and 32 cases returned to “full function” within 6 months. Thirty out of 37 cases never required painkillers at follow-up. When excluding cases with concomitant conditions that affect mobility, the mean LOAD score at follow-up was “6” for both HIF and HCF cases, with median LOAD scores of “4” and “5”, respectively. These scores are within the mild category (0–10) of LOAD score results and 80 and 75% of all HIF and HCF cases respectively had a LOAD score of <10 at follow-up (18). Dogs with no clinical mobility issues may have LOAD scores above zero because LOAD is not a screening tool for the presence of mobility issues but was designed as a disease-severity tool for dogs with diagnosed joint disease (John Innes, personal communication). The LOAD score has been validated as a tool to evaluate canine osteoarthritis and correlated well with other chronic pain indices (26–28). In addition, criterion validity was demonstrated for LOAD with a significant correlation with peak vertical force as measured by a force platform (28).

The decision to surgically treat a case of clinically silent HIF is complicated by the incidence of major complications following transcondylar screw placement. Surgical management of HIF has been associated with higher postoperative complication rates compared to other orthopedic surgeries and the potential need for further surgery has always been a concern when discussing prophylaxis of clinically silent HIF against further fracture (13–15, 29, 30). Even after identifying risk factors associated with higher post-operative complications, such as direction of screw placement, the rate of major complications following transcondylar screw placement remains higher than other orthopedic surgeries with reports of major complication rates of ~20% (13, 16, 31). The most frequent complication necessitating further surgery is deep SSI (13, 14). Therefore, a reduction in the incidence of post-operative SSI would reduce the incidence of major surgical complications. In the first report of HIRS, the total complication rate (major and minor) was 15% for HIF surgery and 29% for HCF repair (17). SSI in that report occurred in one HIF case (3%) and resolved with medical management. Three cases of HCF developed SSI (21%), with one (7%) responding to medical management and two (14%) requiring surgical management. This lower rate of SSI requiring surgical management demonstrated with the HIRS may reduce the concern regarding surgical management of HIF. Prophylactic post-operative antibiotics were prescribed for most cases in that paper, which may have contributed to the low SSI rates. In our study, no cases of SSI were reported by owners in the long-term.

Two cases were reported to have a gradual return of lameness beginning at least 12 months after surgery. These were two of four cases with documented concomitant MCD, which was not surgically managed at the time of HIF surgery. One of these dogs had been euthanized for reasons unrelated to lameness by the time long-term follow-up was performed for this study. The other dog had clinical signs more consistent with osteoarthritis than with implant failure, e.g., post-rest stiffness and lameness that did not deteriorate during activity. Studies have demonstrated that 26–44% of elbows with HIF also have concomitant MCD, and that osteoarthritis is frequently seen in association with MCD (21, 32–34). In the authors' experience, clinical signs of transcondylar screw failure are typically similar to those of HIF at first presentation. That is lameness that becomes worse during activity and improves with rest. However, transcondylar screw failure cannot be excluded and these owners declined further investigation.

The main limitation of this study is the relatively small number of cases. The HIRS is a relatively recently released implant, so it was not widely used some years ago, therefore the case population available to assess long term outcomes is small. Retrospective studies, such as this one, are at risk of aspects of selection bias. However, as most cases were logged prospectively, and the HIRS implant was readily identifiable on the practice management system, the authors are confident that all dogs that were treated with HIRS were identified. Outcomes reported are based on owner-assessment, which might not capture aspects identifiable on veterinary examination and lacks the objectivity of gait analysis tools. However, owners have been documented to over-report complications compared to veterinary professionals and client satisfaction and perception of treatment outcomes are an important consideration when making clinical decisions (35). The definition of successful treatment of HIF differs depending on the reported outcome measure, with some reports focusing on ameliorating pain and lameness, whereas others concentrate on assessing bone healing. Transcondylar screws have been reported to fail at follow-up times of 30 and 36 months post-operatively, which is greater than the follow-up times reported in the present study (36). Analysis of the failed transcondylar screws explanted from these cases revealed a pattern of multidirectional fatigue failure, which may indicate that intercondylar instability persists following screw fixation in the absence of boney union. Walton and others documented at least a partial degree of HIF healing in all cases that were treated using the HIRS at a median follow-up time of 461 days (17). Given the lack of imaging follow-up in this study, we cannot comment upon boney healing in this cohort. Further studies conducting advanced imaging at long-term follow-up would give greater insight into the healing rate of HIF following treatment with HIRS, and this would be an ideal subject of further research.

Overall, taken together with the first report on short-term outcomes of the HIRS, these two studies performed thus far support the use of the HIRS implant for management of HIF and HCF. Twenty-five out of 26 HIF cases reached long-term follow-up with the HIRS implant in place, with 24 cases regaining full function of the limb and 19 never requiring analgesia in the long-term. Two cases of HIF, with concurrent MCD, had a gradual return of lameness beginning over 12 months post-operatively. Eleven out of 14 HCF cases reached long-term follow-up, with all cases returning to full function of the limb and 10 cases never requiring analgesia in the long-term. Owners did not report any occurrence of complications in the long-term in HCF cases.

- I—HIRS; Fusion Implants, Liverpool, UK.
- II—Demineralized bone matrix putty; Veterinary Tissue Bank, Wrexham, UK.
- III—LEAP; Fusion Implants, Liverpool, UK.
- IV—Petalexin; MiPet, Diss, UK.

## Data availability statement

The raw data supporting the conclusions of this article will be made available by the authors, without undue reservation.

## Ethics statement

The animal studies were approved by CVS Ethics Review Board; ethics@cvsvets.com. The studies were conducted in accordance with the local legislation and institutional requirements. Written informed consent was obtained from the owners for the participation of their animals in this study.

## Author contributions

RH: Data curation, Formal analysis, Investigation, Methodology, Writing–original draft, Writing–review & editing. MW: Conceptualization, Supervision, Writing–review & editing. JI: Supervision, Writing–review & editing.
