# Supplementary material for: Long term outcomes of the Humeral Intracondylar Repair System for management of canine humeral intracondylar fissures and humeral condylar fractures
Source: Front Vet Sci. 2024 Jan 3;10:1296940. doi: 10.3389/fvets.2023.1296940 (PMC10793381; doi:10.3389/fvets.2023.1296940)
Supplement: Supplementary file 1 [file Data_Sheet_1.PDF]

CHESTER GATES VETERINARY SPECIALISTS

Elbow Surgery Follow-up Questionnaire

Long term outcomes of the Humeral Intracondylar Repair System for management of Humeral Intracondylar Fissures and Humeral Condylar Fractures

* Required

Dear Owner,

Thank you very much for contributing to this study and for completing this questionnaire. The information you provide will enable us to review the outcomes of patients following elbow surgery using the HIRS implant in dogs.

It should take no longer than 5-10 minutes to complete this questionnaire.

Individual privacy will be maintained in all published and written data from the study.

Yours sincerely,

Rebecca Hood *BVSc MRCVS*
Ben Walton *BVSc DSAS(Orth) MRCVS*

Research Ethics Approval Number: CVS-2021-014

1

Please enter your unique case reference number, found at the top of your invitation letter. *

Complications

This section of questions gathers information on any complications associated with your dog's surgery. We are interested in assessing the progress of dogs over one year after the surgery. Therefore, the following questions are all mainly aimed at assessing the progress of your dog one year post-operatively.

2

Did your dog suffer from any of the following complications in the first 12 months after surgery? *

- [ ] No complications
- [ ] Bruising
- [ ] A fluid filled swelling, which resolved on its own without treatment
- [ ] A fluid filled swelling, which required treatment to resolve (e.g. drainage)
- [ ] Breakdown of the wound, which required intervention (e.g. re-stitching)
- [ ] An infection, which resolved with antibiotic treatment
- [ ] An infection, which required surgical treatment (e.g. removal of implants, amputation)
- [ ] Failure of the implants, which required further surgery (e.g. removal or replacement of implants, amputation)
- [ ] Other

3

If your dog suffered from any complications, please state at what time after surgery the complication occurred. *

- ◯ No complications occurred
- ◯ Within 3 months of surgery
- ◯ 3-6 months after surgery
- ◯ 6-12 months after surgery

4

After the first 12 months post-surgery, did your dog suffer from any of the following complications? *

- ☐ No complications occurred 12 months post surgery
- ☐ An infection, which resolved with antibiotic treatment
- ☐ An infection, which required surgical treatment (e.g. removal of implants, amputation)
- ☐ Failure of the implants, which required further surgery (e.g. removal or replacement of implants, amputation)
- ☐ Sudden return of lameness
- ☐ Gradual return of lameness

Mobility

This section of questions gathers information regarding your dog's current mobility.

5

Does your dog suffer from any other medical or orthopaedic conditions which could affect their mobility or ability to exercise? E.g. heart disease, hip dysplasia etc *

- ◯ Yes
- ◯ No

6

If you answered yes to the above question, please give details of their condition below.

7

How does their current mobility compare to before surgery? *

- ◯ Better
- ◯ Equal
- ◯ Worse
- ◯ Much worse

8

Did your dog return to full (normal, pre-injury) use of the affected leg without any lameness/limping at any point after the surgery, and when? *

- ◯ Yes. Less than one month post-surgery
- ◯ Yes. Between one and three months post-surgery
- ◯ Yes. Between three and six months post-surgery
- ◯ Yes. More than six months post-surgery
- ◯ They never regained full function of the affected leg

9

Does your dog require any painkillers to manage pain associated with the affected leg? *

- ◯ Never
- ◯ Less than once monthly
- ◯ Multiple times a month but not every week
- ◯ Multiple times a week but not every day
- ◯ Every day
